# Therapeutics

**Published:** 1880-05

**Authors:** 


### THERAPEUTICS.

TANNATE OF PELLETIERIN AS A TÆNIAFUGE.—The active principle of the bark of the *Punica granatum*, discovered by M. Tauret, and described by him under the name of pelletierin, has been used with success as a remedy for tænia. It is given in the form of tannate, in doses of (gr. viij) 50 centigrams, followed in two hours by (℥j) 30 grams of castor oil. Dr. Laudrien describes its action as follows: In the two cases observed neither colic nor headache were occasioned. In the first the patient had been prepared by dieting, and a single dose sufficed to expel the tænia entire. In the second, though one dose did not suffice, the patient was not weakened by the treatment. The patient did not exhibit that repugnance so constantly shown to the administration of kousso or pomegranate bark. In a third case the administration of a dose of the hydrochlorate of pelletierin brought away 15 metres of worm, the only phenomena observed being diplopia and a slight tendency to syncope, both of which soon passed off. The pulse and temperature were not influenced by this medicine; nor were the kidneys in any way affected. The medicine seemed to have an elective and toxic action operating solely on the tænia. (*Jour. de Méd. de Bordeaux*, 18th June, 1879; *The New York Med. Record*, 6th Sept. 1879).—*The Practitioner*, Feb., 1880.

RHUS TOXICODENDRON.—Dr. John A. Henning (*Cincinnati Lancet*) calls attention to the value of the above named drug. It admirably meets certain indications. Thus it removes the dull, heavy, aching pain above the eyes in cases of catarrh. It speedily modifies the burning pain of erysipelas. Combined with gelseminum it increases its power to reduce temperature. In forms of inflammation accompanied by a burning, stinging pain, it quickly removes the pain. In spinal irritation, with enfeebled circulation or spinal anæmia, it is an excellent remedy combined with nux vomica. It is also indicated in passive congestion of the brain or in meningitis. Its general action, when taken in proper doses, is as a laxative, diaphoretic, diuretic, and particularly as a stimulant of the nervous system. The dose is from five to fifteen minims in half an ounce of water.—*The Detroit Lancet*, January, 1880.

HYDROFLUORIC ACID INHALATIONS IN DIPHTHERIA.—Bergeron treated diphtheria in twenty-four cases, with five deaths. The dose is one grain for each cubic meter which the apartment contains, evaporated in the space of three hours. He claims (1.) Inhalations thus given never produced injurious results; (2.) All submitted to this treatment for forty-eight hours were cured; (3.) The membranes do not persist beyond the fifth day; (4.) In no case does paralysis supervene.—*Medical and Surgical Reporter*, 3d January, 1880.

